# A natural user interface to integrate citizen science and physical exercise

**DOI:** 10.1371/journal.pone.0172587

**Published:** 2017-02-23

**Authors:** Eduardo Palermo, Jeffrey Laut, Oded Nov, Paolo Cappa, Maurizio Porfiri

**Affiliations:** 1 Department of Mechanical and Aerospace Engineering, New York University Tandon School of Engineering, Brooklyn, NY, United States of America; 2 Department of Mechanical and Aerospace Engineering, “Sapienza” University of Rome, Rome, Italy; 3 Department of Technology Management and Innovation, New York University Tandon School of Engineering, Brooklyn, NY, United States of America; Universidad Europea de Madrid, SPAIN

## Abstract

Citizen science enables volunteers to contribute to scientific projects, where massive data collection and analysis are often required. Volunteers participate in citizen science activities online from their homes or in the field and are motivated by both intrinsic and extrinsic factors. Here, we investigated the possibility of integrating citizen science tasks within physical exercises envisaged as part of a potential rehabilitation therapy session. The citizen science activity entailed environmental mapping of a polluted body of water using a miniature instrumented boat, which was remotely controlled by the participants through their physical gesture tracked by a low-cost markerless motion capture system. Our findings demonstrate that the natural user interface offers an engaging and effective means for performing environmental monitoring tasks. At the same time, the citizen science activity increases the commitment of the participants, leading to a better motion performance, quantified through an array of objective indices. The study constitutes a first and necessary step toward rehabilitative treatments of the upper limb through citizen science and low-cost markerless optical systems.

## Introduction

Citizen science empowers people with little to no scientific training to participate in research led by professional scientists in different ways [1, 2]. The benefit of such an activity is often bidirectional [3], whereby professional scientists leverage the effort of a large number of volunteers in data collection or analysis, while the volunteers increase their knowledge and get the satisfaction of taking part in a scientific endeavor. A well-known example is Galaxy Zoo [4], a citizen science astronomy project that involves people in the classification of a large number of images obtained through several astronomic surveys. To date, volunteers have provided millions of classifications, enabling the development of improved classification and anomaly-detection algorithms for future surveys [5]. Citizen science is not limited to online projects, but also may involve the physical participation of volunteers [6–11].

In some cases, citizen science provides an additional benefit, such as increasing awareness of social and environmental issues [12]. In Gowanus Voyage [13] (part of the citizen science project Brooklyn Atlantis [14]), volunteers were part of the environmental monitoring of one of the most polluted bodies of water in the United States, the Gowanus Canal in Brooklyn, NY [15]. Driving miniature sensor-equipped boats via remote control in the canal, volunteers contributed to mapping the water quality, while learning from scientists about the pollution of a body of water flowing through the highly populated urban area where they lived.

The wide spectrum of motivational factors tapped by citizen science [16–18] may be applied to other settings such as neuro-rehabilitation. Certain rehabilitative regimens may consist of regularly performing repetitive exercises [19], and adherence to these regimens is crucial for the effectiveness of the therapy [20]. Although a number of “serious games” has been recently explored for rehabilitation purposes [21], their use in long term rehabilitation treatments has been often questioned, especially with respect to elderly patients [22, 23]. Such a population was found to prefer the inclusion of content that has the potential to stimulate reflection and problem solving activity [24].

The possibility of engaging participants in science learning while performing rehabilitative exercises has been investigated in [25]. In that study, healthy adults who navigated a virtual environment using a low-cost haptic joystick attained higher levels of satisfaction when exposed to science content. A more recent effort [26] demonstrated that contributing to a citizen science project through the same low-cost device could provide an additional stimulus for children with hemiplegia. The study indicated that conducting a scientific task represented a more engaging means to perform the protocol, compensating for the tediousness of repetitive exercise schemes.

Low-cost haptic devices, such as the Novint Falcon used in these studies [25, 26], afford the administration of force feedback—the application of a contact force to the user’s hand based on a pre-programmed strategy [27–30]. The employment of such a technology, typical of robot-mediated therapy (RMT) devices [31–37] in a domestic environment, represents a promising opportunity for long-lasting treatments [38–42]. However, this portability is accompanied by a limited workspace, which hampers the possibility to perform large movements needed for some treatments [43]. To address these drawbacks, consumer motion systems have been recently proposed for human analysis and rehabilitation [44].

The Microsoft Kinect sensor, for example, is a markerless human motion tracker capable of estimating three-dimensional (3D) coordinates of human joints in a skeleton model [45]. Initially designed for gaming, it has been repurposed as an input device for natural user interfaces [46], where the movement of the human body, or a part of it in the form of gestures [47], is integrated in human-computer applications [48]. Such a functionality stimulated the use of Kinect both in computer-based rehabilitation [49, 50] and human motion analysis [51]. Importantly, Fernandez-Baena et al. [52] have demonstrated that the Kinect yields joint angles highly comparable to those identified by professional-grade optoelectronic systems, validating its use for objective evaluation of physical exercise.

The present study is driven by the overarching research goal of integrating the motivational factors of citizen science in physical rehabilitation activities. In particular, this study builds on our previous studies, by exploring the possibility of improving engagement in upper limb rehabilitation through citizen science participation and haptic devices in [25] and [26]. In contrast with these efforts, we shift from a laboratory or clinical setting to directly involve participants in environmental data collection. This study represents the first step towards this goal, where we test its usability on healthy participants.

Specifically, we seek to demonstrate the possibility of integrating Kinect-based physical activities and citizen science. The citizen science task was based on Gowanus Voyage, and consisted of water quality mapping of a polluted body of water by means of a sensor-equipped miniature boat. To evaluate the benefit of the physical exercise toward citizen science, we assessed whether the motion commonly involved in rehabilitative exercises represented an engaging and effective means to control the instrumented boat with respect to a traditional system. To evaluate the benefit of citizen science on physical performance, we tested if a better motion performance of participants was associated with a better performance on the environmental task, and whether participation in this scientific project enhanced engagement and physical performance.

## Materials and methods

The entire study, along with the consent procedure, was approved by the NYU Institutional Review Board (IRB). Participants gave their written informed consent.

### Experimental setup

**Fig 1:**
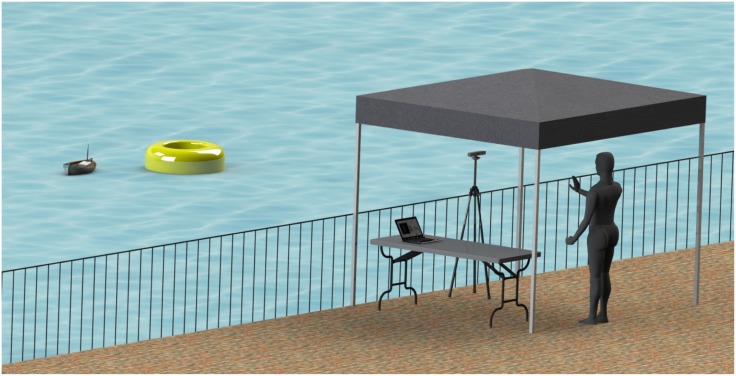
Schematic of the experimental setup. The participant performed the trial on the canal bank. A tent reduced the sunlight exposition of the Kinect to reduce artifacts. The gestures captured by the Kinect were processed in real time by the software running on a laptop and the corresponding commands were sent to the boat via a radio connection. At the same time, water quality data were transmitted from the boat to the laptop.

In Fig 1, we display a schematic of the experimental setup, consisting of a laptop computer, a Microsoft Kinect sensor (release 1.0, Microsoft, Redmond, WA, USA), and a miniature boat instrumented with water quality sensors, a GPS unit, and a radio module. The boat was controllable in thruster speed and rudder angle up to a distance of about 100m, and equipped with an Arduino microcontroller. The microcontroller was programmed to collect GPS and water quality data (conductivity, pH, temperature, and dissolved oxygen) and transmit the information to a base station via the radio module. The Arduino received driving data (thruster speed and rudder angle) via the radio module to control the DC motor connected to the thruster and the rudder servomotor.

The base station, where the subjects performed the experimental session, was located on the canal bank. It included a laptop computer and the Kinect sensor. The Kinect fused data-streams from two cameras: a VGA resolution RGB camera and an infrared camera. An additional linearly polarized filter film was placed on the infrared camera for using the Kinect outdoors. The experimental sessions were conducted underneath a 2.44 × 3.04 m tent with one side open to the canal, to block direct sunlight glare on objects within the sensor range, and permit a clear recognition of the subject by the Kinect.

A National Instruments LabVIEW software (release 12.0, NI, Austin, TX, USA) was developed and run on the laptop to: acquire 3D coordinates of the body joints from the Kinect at a frequency of 30 samples per second; log data to a binary file; compute and send driving commands to the boat based on predefined gesture; receive water quality data from the boat; and provide a driving interface feedback on the thruster speed and rudder angle levels to the participant, through virtual analog gauges. In our experiments, we found that the acquisition rate of the Kinect was 30.05±1.74 frames per second, different from [53], in which a larger variation in the sampling rate was documented. The software was developed using the “Kinesthesia” NI LabVIEW Programming APIs for Microsoft Kinect SDK. The Kinect SDK allows for setting the smoothing parameters for the 3D joint position estimation [54]. Based on pilot experiments, we chose the following parameter set to adequately estimate joint positions in outdoor environments: {Smoothing = 0.5, Correction = 0.1, Prediction = 0.5, JitterRadius = 0.1, MaxDeviationRadius = 0.1}.

The experimental protocol and the data processing were designed around the capabilities of the Kinect [52, 55]. For example, Obdrzalek et al. [55] evaluated the accuracy of the Kinect in localizing human joint centers and demonstrated the primary role of both the subject view angle from the Kinect sensor and the subject posture (sitting or standing). The highest accuracy was attained for the subject standing in frontal view, with a maximum localization error of approximately 70 mm for the wrist joint. The error in the estimation of the joint angles was assessed by Fernandez-Baena et al. [52], during dynamic trials, and a maximum error of 16% of the range of motion (ROM) was found for the shoulder motion in the frontal plane.

Resting on this evidence and a few pilot experiments, the participant was positioned in front of the Kinect sensor (placed on a tripod at a height of 155 cm) at a distance of approximately 2 m. The participant was instructed to move only their dominant arm, without constraining the other arm, and not to move their legs and feet, unless a fall was imminent. An arm gesture in front of the Kinect was used to drive the boat tasked with completing a path in the minimum possible time. This type of gesture was designed to push the subject to extend their arm, keeping it far from the trunk. A widely adopted typology of rehabilitative exercises in hemiplegia is based on the repetitively performing simple movements with the affected arm, in some cases constraining the less affected one [56, 57]. Here, by lifting up their dominant arm, the participant engaged the control, and the software interface began indicating the thruster speed and the steering angle as the driving feedback.

**Fig 2:**
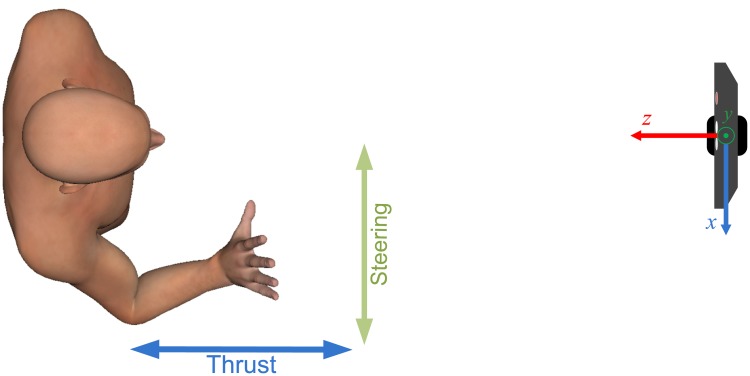
Schematic of the driving gesture implemented (top view). Moving the wrist forward and backward controls the thrust of the boat, while the left-right movement controls the steering angle. On the right, the Kinect reference frame is depicted.

Moving the wrist forward and backward in the horizontal plane allowed the participant to regulate the thruster speed, while the left-right motion of the wrist in the horizontal plane enabled them to set the rudder angle. In particular, referring to the Kinect reference axes shown in Fig 2, the input value of the thruster speed was proportional to the *z*-component of the vector joining the wrist and the hip center, divided by the arm length (from the shoulder joint to the wrist). Similarly, the input value for the rudder angle was proportional to the *x*-component of the above mentioned vector, divided by the arm length. In this way, the control parameters were independent of the participant’s position with respect to the sensor or their arm length.

### Experimental protocol

**Fig 3:**
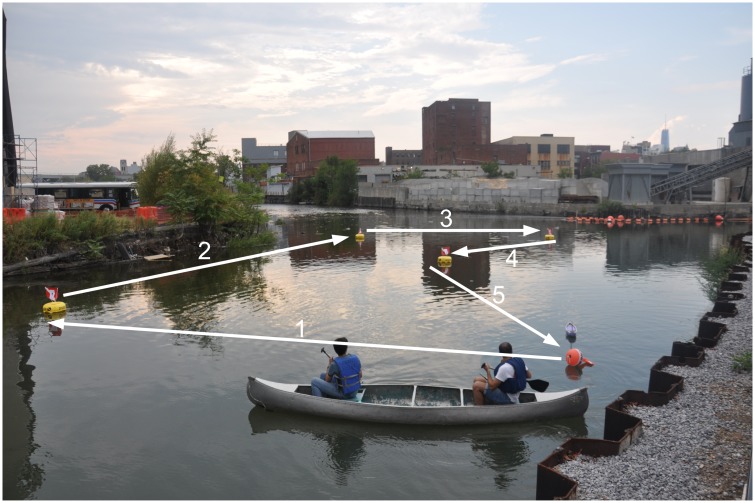
The challenge path. The orange buoy represents the starting and the finish of the path, performed driving the boat around four numbered yellow buoys.

To examine the viability of the platform, all subjects were instructed to drive the boat along the same predefined path marked by a set of five buoys in the shortest time possible, and to avoid deviations from the path. Since each participant controlled the same boat along the same path, the driving performance of subjects was assessed by measuring the lap time with a stopwatch. Fig 3 shows the path obtained by researchers, by placing numbered buoys in the canal using a canoe. The individual in this manuscript has given written informed consent (as outlined in PLOS consent form) to publish these case details. The path was traversed starting from the orange buoy, turning around the yellow buoys following the numbered order, and coming back to the starting place. Each participant performed only one trial, and only trials in which the complete path was followed were considered in the study. None of the participants experienced fatigue by the end of the trial.

A total of 60 healthy adults (46 male and 14 female) were recruited to participate in the experiments, among pedestrians passing by the canal bank. Only participants with no prior experience with Brooklyn Atlantis project were considered eligible for the study. The age range of participants was 18–50 years, as within such a range, the kinematic behavior of the upper limb is comparable across subjects [58]. The participants involved were divided into three groups of equal size, described below and classified in Table 1. The inclusion of subjects in the groups was randomized.

**Table 1:** Classification of the three experimental groups.

Group	Control device	Driving feedback	Awareness of scientific task	Enviromental data feedback
J	Joystick	No	No	No
KNC	Kinect	Yes	No	No
KC	Kinect	Yes	Yes	Yes

To evaluate the effectiveness of the entire Kinect-based platform developed as means for helping to collect environmental data, one of the three groups drove the boat along the path using a traditional joystick (group J), with a trigger to control the thruster and a knob for the steering. The other two groups used a boat controlled via the Kinect (group KC and group KNC), differentiated as described in the following. To test the effect of the scientific research context on motion performance, the KC group participants were informed at the beginning of the trial that their effort will be used to automatically collect data from water quality sensor, similar to data collected by scientists and included in the Brooklyn Atlantis project. At the end of the trial, the KC group participants saw the result of this collection as a screen pop-up with the map of the canal with water quality values superimposed, the mean values of the collected data, and a brief explanation of their meaning. The participants of the KNC group were told that their trial was only targeted at testing their driving skills and assessing the platform and no environmental data collection report was shown at the end. During the trials, kinematic data of subjects in KC and KNC were recorded to evaluate the quality and quantity of the body motion through the motion performance indices detailed below.

At the end of the session, participants were asked to fill out a survey, described in the next subsection. The survey consisted of ten items (Q1–Q10). The first two items were about the controlling device they used (joystick or Kinect) and their gender. The third asked participants if they were a registered user on the Brooklyn Atlantis website [59] to test their eligibility in the study. The remaining items are reported below. The first four (Q4–Q7) were intended to rank the engagement of the participants and the perceived maneuverability of the control strategy they used. The last three items were dedicated specifically to the group involved in the data collection (KC), to evaluate their interest in the scientific activity. For each item the participants chose a level of agreement using a four point Likert scale [60] (strongly agree, somewhat agree, somewhat disagree, and strongly disagree). We used a four point scale with no neutral option to push participants to think about the question before answering, as the neutral option is often chosen from indecision [61].

Q.4 Today’s activity was enjoyable.
Q.5 It was easy to control the boat.
Q.6 It was fun to control the boat.
Q.7 The boat moved as I intended it to.
Q.8 I am helping scientists.
Q.9 I am contributing to environmental monitoring.
Q.10 I have learned much about pollution and recovery of the Gowanus Canal today.

We assigned an ordinal values scale from 1 to 4 to the answers of survey items Q4–Q10, increasing with the level of agreement. The scores obtained for the answers to items Q4 and Q6 should be related to the level of engagement perceived during the experience, and they were summed to yield the “engagement factor”, ranging from 2 to 8. A higher score should correspond to a higher level of engagement of the participant. Similarly, answers Q5 and Q7 were combined to yield the overall “maneuverability factor”, ranging from 2 to 8, where a higher score should indicate that the subject felt the boat easier to control. Survey items Q8, Q9, and Q10 concerned the interest of the subject in the scientific task and their feeling to be an active part of it. The scores obtained for these answers were summed to gauge the interest of the participants toward the citizen science project, obtaining a total score ranging from 3 to 12.

### Data processing

**Fig 4:**
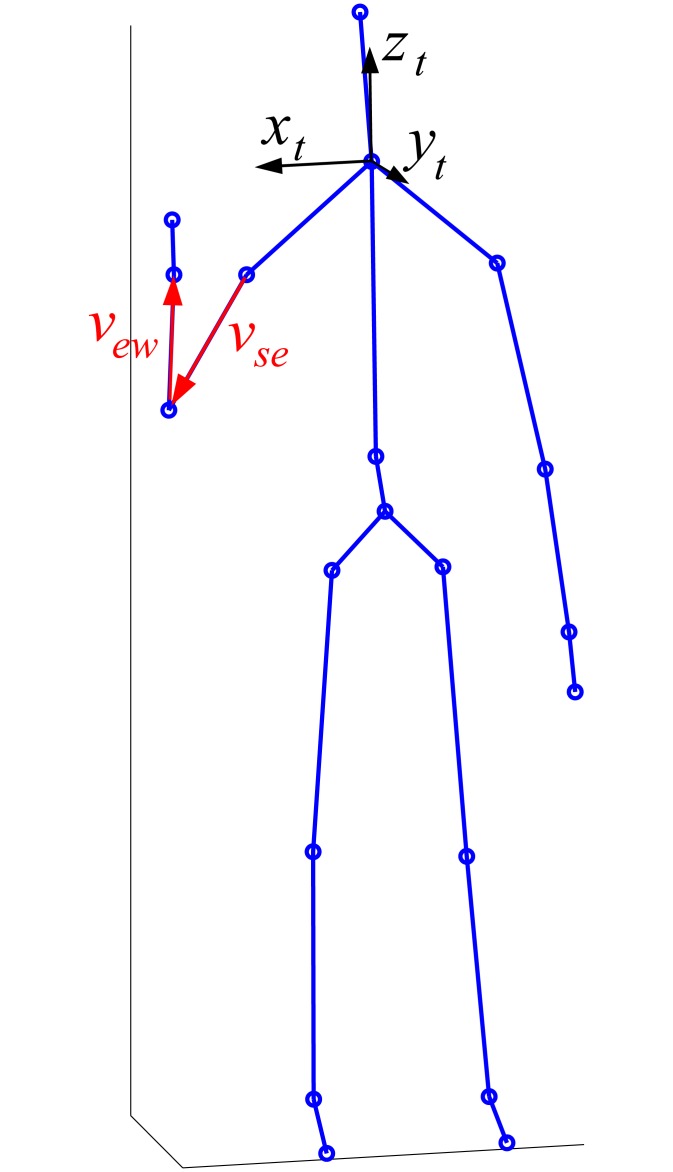
Schematic of the kinetic model of the human body obtainable with Kinect data. The circles represent the 20 articular joint positions provided by the Kinect system. The local coordinate system of the trunk is depicted in black and *v*_*se*_ and *v*_*ew*_ are the vectors connecting the shoulder joint with the elbow joint and the elbow joint with the wrist joint, respectively.

Using the 3D coordinates of the 20 joint centers provided by Kinect, a kinematic model of the human body can be built [54]. Fig 4 shows a schematic of the model reconstructed in MATLAB (release 7.12, The MathWorks, Natick, MA, USA) from data of one acquisition frame. The model does not identify three reference points in each body segment, thereby not allowing to solve the complete three-degree of freedom joint angular kinematics. As a result, the model cannot be used to acquire information on some joint angles, such as the arm rotation around its longitudinal axis. For this purpose, each body segment should be assumed to be rigid so that its pose could be computed from the coordinates of at least three points belonging to it. In the Kinect model, only the trunk and the pelvis have more than two measured points. Thus, focusing on the upper limb, it is possible to define two joint rotations for the shoulder and one for the elbow.

We defined the local coordinate system for the trunk with the *z*_*t*_-axis directed parallel to the vector connecting the spine joint with the shoulder center [54], the *x*_*t*_-axis perpendicular to the *z*_*t*_-axis and lying in the plane containing the two shoulder joints and the shoulder center, and the *y*_*t*_-axis following the right-hand rule. The origin of the local coordinate system was located at the shoulder center joint. In such a model, the shoulder motion is approximated through the rotation of the arm with respect to the trunk, without including the motions of two anatomical parts articulating in between them, the scapula and the clavicle [62]. We computed the two joint angles of the shoulder and joint angle of the elbow following Pacilli et al. [63]. With reference to Fig 4, the angle formed by the vector connecting the shoulder center and the elbow center (*v*_*se*_) with the *z*_*t*_*x*_*t*_-plane of the trunk is termed as shoulder flexion/extension (Fl/Ex). The angle that the projection of *v*_*se*_ on the *z*_*t*_*x*_*t*_-plane of the trunk forms with *z*_*t*_ is the shoulder abduction/adduction (Ab/Ad). Finally, the elbow Fl/Ex is the angle between *v*_*se*_ and the vector connecting the elbow and the wrist joint centers (*v*_*ew*_).

A set of kinematic performance indices were selected as a tool for evaluating motor skills. These indices were chosen in terms of quantity and quality of the motion of the participant, as both these factors are considered crucial in quantifying effectiveness of physical rehabilitation [64, 65]. The following parameters were considered:

1. **ROM** of shoulder and elbow, defined as the difference between the maximum and the minimum level of the angle reached during the trial. In particular, for each trial three values of the ROM were computed (shoulder Fl/Ex, shoulder Ab/Ad, and elbow Fl/Ex).
2. **Root mean square of angular velocity (AVRMS)** of shoulder and elbow. The values of this new index were calculated as the root mean square of the derivative of the three computed joint angle signals. AVRMS is intended to quantify the peak-to-peak variation of joint angular velocity.
3. The **planarity motion index (PMI)**. We defined this index as
  $$\frac{\sigma_{c}}{\sqrt{\sigma_{a}\sigma_{b}}}$$ (1)
  where *σ*_*a*_, *σ*_*b*_, and *σ*_*c*_ were the three eigenvalues, in descending order, of the 3 × 3 covariance matrix from the three components of the trajectory of the wrist in the *xyz*-coordinate system. A value close to 0 implies that the motion of the wrist is 2D as requested by the planar arm motion of the driving task. 3D motions revealed by values of PMI closer to 1 should be associated with low motion control levels.
4. **Trunk stabilization index (TSI)**, defined as the standard deviation of the time traces roll and pitch angles of the trunk during the trial, considering the roll-pitch-yaw rotation sequence of the *x*_*t*_*y*_*t*_*z*_*t*_-coordinate system with respect to the *xyz* one. This metric scored the capability of the subject to isolate the gesture to the arm level without losing balance.
5. The **speed metric (SM)**, measured as the mean velocity of the wrist above a threshold value divided by the peak velocity, represents a score of the smoothness of the motion [66].
6. The **normalized number of submovements (NNSM)** is a modified version of the number of submovements (NSM) [67], obtained by dividing the NSM by the time duration of the trial in seconds.

As a first step in the data analysis, we sought to verify if the Kinect platform represents a valid solution for performing water quality data collection. Specifically, we evaluated the solution both from a subjective and an objective point of view. To assess the statistical significance of the collected survey measures, we used the chi-square test [68], evaluating if the obtained score distributions for engagement factor and maneuverability factor in the three groups were different from an expected value due to chance. The chi-square test was also used to assess differences in engagement factor and maneuverability factor between groups.

Beyond the subjective evaluation provided by the survey, we considered the completion time of the task as an objective measure. In particular, we analyzed differences among the three groups with a Mann-Whitney U test [69], as the measurement sample was non-normally distributed, as seen from a Kolmogorov-Smirnov test [70] (*p* < 0.001).

To evaluate the validity of the scientific task as a physical exercise, we looked for a correlation between physical performance and skill in controlling the boat. Such a relationship is important to predict whether future improvement of subjects in their driving skills, due to continuous practice over time, could be associated with an improvement in their motion quality. Only kinematic data of the targeted body segments from the two Kinect groups (KC and KNC) were used, since no rehabilitative gesture was performed by the joystick group (J). The Pearson’s correlation coefficients (and probability levels associated) were calculated between the time of completion for all the trials of both the Kinect groups, and the corresponding performance indices.

A chi-square test was executed on the interest score obtained for the KC participants to test whether their response was different from chance. Finally, a one-way ANOVA, with the data collection (KC and KNC) as the independent variable and all the motion performance indices as dependent variables was performed. This analysis sought to elucidate the effects of the awareness of the scientific activity.

For all the tests, statistical significance level was set at *p* = 0.05.

## Results

### Participants from all groups felt engaged

Participants considered the two control strategies to be highly engaging, and no differences were found between them. Specifically, the total score for the engagement factor was higher than the neutral level of 100 for the three groups, being very close to the maximum level of 160 with a distribution of the answers different from chance (J: *χ*^2^(6) = 188.667, *p* < 0.001; KC: *χ*^2^(6) = 188.667, *p* < 0.001; and KNC: *χ*^2^(6) = 213.067, *p* < 0.001) as shown in Table 2. The engagement factor values of the three groups were indistinguishable (J vs. KC: *χ*^2^(6) = 0, *p* = 1; J vs. KNC: *χ*^2^(6) = 0.23, *p* = 0.999; and KC vs. KNC: *χ*^2^(6) = 0.23, *p* = 0.999).

**Table 2:** Engagement factor, maneuverability factor, and lap time for the three groups. Groups include participants controlling the boat via the joystick (J), the Kinect in data collecting trials (KC), and the kinect in non-collecting trials (KNC). For engagement and maneuverability factors, the reported values represent the total score obtained from all subjects. For the completion time, instead, mean values (standard deviation) are reported. Values sharing the same superscript in the same column are not statistically different.

Group	Engagement factor	Maneuverability factor	Lap time (s)
J	155^a^	155^a^	74 (26)^a^
KC	155^a^	131^b^	101 (38)^b^
KNC	156^a^	123^b^	119 (89)^b^

### Both the joystick and the Kinect were effective for controlling the boat

As shown in Table 2, participants in each of the three groups perceived the boat to be maneuverable, whereby the total score was higher than the neutral value of 100 with a distribution of the answers different from chance for all three groups (J: *χ*^2^(6) = 212.667, *p* < 0.001; KC: *χ*^2^(6) = 24.667, *p* < 0.001; and KNC: *χ*^2^(6) = 20.667, *p* = 0.002). In addition, participants felt that the boat was more maneuverable with the joystick than the Kinect (J vs. KC: *χ*^2^(6) = 19.9, *p* = 0.003 and J vs. KNC: *χ*^2^(6) = 26.22, *p* < 0.001). No difference was found due to the awareness of the scientific task (KC vs. KNC: *χ*^2^(6) = 2.22, *p* = 0.894). As evidenced in Table 2, Kinect users completed the path in a longer time than remote control users (J vs. KC: U = 99.50, *p* = 0.007 and J vs. KNC: U = 129.00, *p* = 0.055). No difference in the time completion was noted between the two Kinect groups (KC vs. KNC: U = 180.00, *p* = 0.588). The minimum trial duration was 51 s and the maximum was 411 s.

### Motion performance was correlated with the time of completion of the task

**Fig 5:**
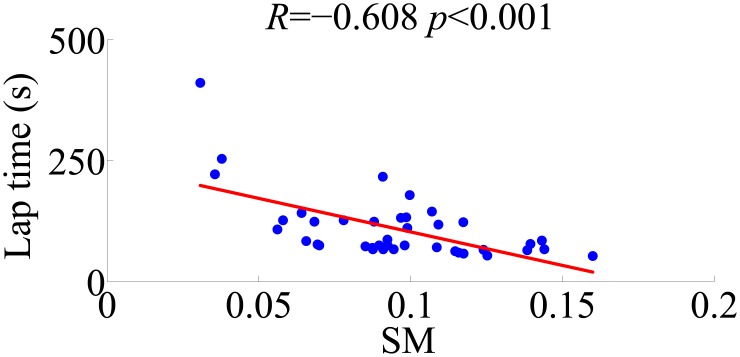
Scatter plot of the completion time of the trial with respect to the speed metric (SM) scores. The regression line is depicted in red. The Pearson’s correlation coefficient (*R*) and the probability associated (*p*) are reported above.

Most of the proposed motion indices were correlated with the time of task completion. Table 3 reports the Pearson’s correlation coefficients, and the associated level of probability, between each of the indices and the lap time. A moderate correlation was found between the ROM of all the considered angles and the driving performance (shoulder Fl/Ex: *R* = 0.532, *p* < 0.001; shoulder Ab/Ad: *R* = 0.512, *p* = 0.001; and elbow Fl/Ex: *R* = 0.512, *p* = 0.001). A correlation was found for AVRMS of elbow Fl/Ex (*R* = 0.341, *p* = 0.031). The TSI showed a weak correlation for the roll plane (*R* = 0.392, *p* = 0.012), and a moderate correlation for the pitch plane (*R* = 0.500, *p* = 0.001). A strong negative correlation was found for the SM index (*R* = −0.608, *p* < 0.001), displayed for clarity in Fig 5. No significant correlation, instead, was found for the AVRMS of the two shoulder joint angles (Fl/Ex: *R* = 0.231, *p* = 0.152; Ab/Ad: *R* = 0.138, *p* = 0.394), for the PMI (*R* = 0.124, *p* = 0.444) and for the NNSM (*R* = 0.040, *p* = 0.807), as reported in Table 3.

**Table 3:** Correlation coefficients and associated probability values between the time of completion of the task and the motion performance indices.

Index			*R*	*p*
**ROM**	Shoulder	Fl/Ex	0.532	<0.001
		Ab/Ad	0.512	0.001
	Elbow	Fl/Ex	0.512	0.001
**AVRMS**	Shoulder	Fl/Ex	0.231	0.152
		Ab/Ad	0.138	0.394
	Elbow	Fl/Ex	0.341	0.031
**TSI**		Roll	0.392	0.012
		Pitch	0.500	0.001
**PMI**			0.124	0.444
**SM**			−0.608	<0.001
**NNSM**			0.040	0.807

### Participants were interested in the citizen science activity

Participants maneuvering the boat via the Kinect who were made aware of the scientific component of the test (KC) exhibited a remarkably high interest in the citizen science project (*χ*^2^(9) = 91.253, *p* < 0.001), reaching a total score of 202 on a range of possible values from 60 to 240, where the neutral value was of 150, with a distribution of the answers different than chance.

### Awareness of the scientific task increased the motion performance

**Fig 6:**
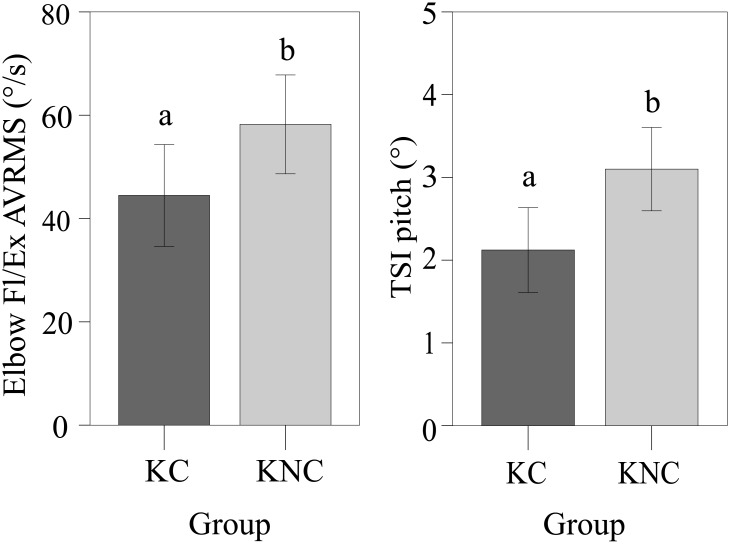
Bar plots of two kinematic indices in the two Kinect groups. Root mean square of the angular velocity (AVRMS) for the elbow Fl/Ex and pitch trunk stabilization index (TSI) in the two Kinect groups (KC and KNC) are depicted. Error bars indicate the 95% confidence interval. Statistically significant differences are indicated with different letters.

Table 4 reports mean and standard deviation values for the indices in the two Kinect groups. ANOVA results showed a significant effect of the awareness of the scientific component on the Fl/Ex AVRMS of the elbow (*F* = 4.901, *p* = 0.033) and on the TSI in the pitch plane (*F* = 7.345, *p* = 0.010), whereby KC participants exhibited a lower mean Fl/Ex AVRMS of the elbow and a higher trunk stability (TSI), as depicted in Fig 6. For all other indices considered, mean values of the groups were not statistically different.

**Table 4:** Mean (standard deviation) values for the motion performance indices among all the subjects of the two Kinect groups. Statistically significant differences are marked with stars (*p*(*) = 0.033, and *p*(**) = 0.010).

Index			KC	KNC
**ROM [°]**	Shoulder	Fl/Ex	81.8 (25.9)	93.0 (23.5)
		Ab/Ad	170.3 (86.8)	226.1 (109.6)
	Elbow	Fl/Ex	98.7 (44.6)	125.5 (38.5)
**AVRMS [°/s]**	Shoulder	Fl/Ex	33.9 (15.7)	41.6 (11.6)
		Ab/Ad	63.8 (35.5)	75.4 (35.8)
	Elbow	Fl/Ex	44.5 (21.1)*	58.2 (20.4)*
**TSI [°]**		Roll	3.9 (1.6)	3.6 (1.3)
		Pitch	2.1 (1.1)**	3.1 (1.1)**
**PMI**			0.32 (0.12)	0.31 (0.12)
**SM**			0.98 (0.22)	0.91 (0.37)
**NNSM [s^−1^]**			0.52 (0.17)	0.55 (0.13)

## Discussion

In this paper, we utilized a new low-cost technology to bridge citizen science and physical exercise, linking two activities that can benefit each other. Specifically, we proposed the use of a Kinect motion system to perform citizen science tasks of environmental data collection through an instrumented boat, during physical exercises tailored for future rehabilitation studies. Our results demonstrate that the use of this off-the-shelf device offers an engaging means to contribute to a scientific task without compromising the maneuverability that could be achieved with a more traditional remote control joystick. The markerless human motion tracker device allows for delivering relevant exercises, which can be characterized by wide and unrestricted 3D movements, while recording the body kinematics in time and scoring the subjects’ motion performance.

Although several Kinect-based strategies have been already established to foster physical exercise [50, 51], their integration in citizen science activity has not been explored. The novelty of our study lies in the original use of Kinect for controlling a mechatronic device that collects data for a scientific cause. Unlike other studies based on motion capture, we leveraged participation in citizen science to strengthen motivational factors in physical exercise. Testing this novel concept required an experimental study to its acceptance by users, whose response to Kinect-based citizen science activities could not have been directly anticipated from the literature on gaming [45–47]. Virtual environments utilized in most gaming-based systems do not present the intricacies that characterize our mechatronic system, such as inertia, communication delays, and unstable or unpredictable behaviors due to interactions with objects. These features might have rendered the proposed solution ineffective or unattractive, thereby requiring a dedicated experimental study, which is at the core of this work. Just as the reception of our technology by users was difficult to predict, the specific role of the motivational aspects of citizen science was also elusive and warranted a focused study.

As hypothesized, the use of an unconventional strategy to drive a radio controlled device was perceived as less maneuverable with respect to a traditional remote control, a perception confirmed by a higher completion time of the task. However, the mean maneuverability was significantly higher than the satisfactory level, suggesting that the opportunity for physical exercise offered by the proposed system does not hinder the efficiency in completing the environmental monitoring task. These positive results suggest that the scientific purpose will not be regarded only as a recreational content, but also as a concrete environmental monitoring activity, an important driver for the success of the platform developed here.

Subjects who displayed a higher motion coordination and body stability were also found to be more efficient in controlling the instrumented boat. This relationship suggests that the awareness of the scientific contribution could lead to higher levels of care and accuracy in the task. The need of further developing this link is strengthened by the observed scientific interest and motion performance of participants aware of the environmental data collection they were contributing to. Participants informed of the scientific value of their effort performed better and indicated a strong interest in the overall citizen science project.

Our results confirm the hypothesis that physical activity involving a set of body motions is perceived as engaging, despite the fact that it may limit maneuverability [71]. It is possible that the gestures performed by the participants could offer an additional motivational driver, similar to instances of gamification in citizen science [72, 73]. Such drivers could thus supplement the intrinsic motivations associated with citizen science [74, 75] through recreational elements that sustain the participants’ involvement. This recreational aspect compensated for the increased difficulty as compared to the regular joystick and, as a result, the participants did not feel the use of gestures to be limiting in the completion of their task. Even if Kinect users performed the task in a time period about 50% longer than those who used the traditional remote control, they felt that the Kinect-based control was effective in maneuvering the boat. The volunteers’ perception of the effectiveness of their effort has often been proposed as a valid indicator of their future contribution to the project [16].

Engagement and maneuverability scores obtained in this study may be considered as predictors for the success of a potential rehabilitation therapy that would leverage citizen science and Kinect. In future applications of the platform, we will enhance objectivity of the assessment of participant interest, beyond survey administration. Through a different design, based on multiple subsequent sessions, we plan to investigate participants’ adherence to the project, overcoming some of the potential limitations of traditional survey instruments [76]. The endurance of participants may be evaluated by the amount that continue to follow the activity to the last sessions, a more representative index of long-lasting interest, free from ephemeral enthusiasm due to curiosity.

Beyond assessing the possibility of obtaining quantitative indices of motion performance via a low-cost device, our study explored the feasibility of a novel type of physical exercise with potential for future rehabilitation applications. Further studies on subjects with pathologies will be grounded on the positive findings of this work, where a correlation between the proposed motion metrics and participants’ ability to complete the path was observed. Such a result offers a promising basis for future efforts to investigate the suitability of the activity tested in this study as a rehabilitation task. Traditional upper limb rehabilitation tasks train patients to directly increase their precision in movements via the execution of a precise arm motion [77, 78]. The subjects of this study, instead, were instructed to drive a miniature boat along a path in the minimum possible time. We expect citizen science activities to increase both engagement and motivation of rehabilitation patients in future application of the proposed platform, expanding on the gaming applications tested herein. Our methodology expands behavioral rehabilitation, by providing an engaging and fun natural user interface, a tangible scientific contribution, and an attractive low-cost markerless technology for human motion capture.

Future efforts on subjects with pathologies will be informed by prior studies which have pointed at the potential viability of using the Kinect for stroke rehabilitation and elder care [50]. Some of these efforts posit that the inclusion of gaming elements [49, 79] and visual feedback [80] may induce higher levels of enjoyment, and consequently, improve patient adherence to rehabilitation regimens. Although these efforts offer compelling evidence in favor of the Kinect, long-term studies necessary for demonstrating its actual effectiveness for motor recovery have yet to be undertaken. Building on the findings of this study, future studies should contrast the proposed platform to both traditional physical rehabilitation and computer-based rehabilitation via the Kinect. Comparing motor recovery in an experimental group that uses the proposed methodology to control groups, employing traditional or computer-based rehabilitation, will shed light on the potential of the Kinect. This comparison will offer evidence for the feasibility of computer-based rehabilitation via the Kinect and help testing whether the motivational factors tapped by our approach would translate into a higher rehabilitation effectiveness.

Our preliminary evidence suggests that motivation of patients to improve their performance in driving the boat could be an additional element to be leveraged toward the delivery of effective rehabilitation treatments. Specifically, the correlation found between ROM of shoulder and elbow angles and the time completion of the task indicates that a smaller and more controlled gesture permits a better control of the boat. Similarly, the correlation between the TSI and the lap time suggests that subjects with a better balance are more skilled in performing the trial. The smoothness of the motion is a further feature that correlates with the lap time, whereby subjects exhibiting higher SM execute the trial in a shorter time. Thus, similar to virtual gaming elements considered in Kinect-based rehabilitation treatments [49, 50, 81], the possibility to remotely drive physical objects might offer an additional venue for improving the delivery of rehabilitation treatments.

Based on our results, the set of selected motion performance indices is expected to represent a valid tool in quantifying the motor deficit on a population with pathology. The correlation of some of these indices with driving capabilities, found on healthy subjects, is predictive of the efficacy of the water quality data collection activity to improve subjects’ motor skills. We would anticipate a comparable scenario from future application of this methodology on a population with motor deficit. The higher variation of motor skills in such a population would probably result in a wider spread of the performance indices values, as patients with upper limb impairment often present limited ROM [64], a reduced stability of the trunk when moving the arm [82], and a lower motion smoothness [66].

In agreement with our expectations, our results indicate that the awareness of the scientific task enhances subjects’ performance. This hypothesis rested upon findings of a previous study, where the inclusion of scientific content was shown to increase the level of satisfaction in a rehabilitation activity based on a virtual environment [26]. Here, we increased the level of commitment of the participants by direct involvement in authentic environmental data collection as part of a citizen science project. The task of following the path was designed to invoke a similar set of predefined movements between the two Kinect groups, regardless of awareness of environmental data collection. Subjects involved in the scientific task exhibited a significantly higher TSI and a reduced ROM of the shoulder Fl/Ex, which are both valid indicators of improved motion performance. It is thus tenable that motivational drivers addressed by citizen science, such as personal interest and the desire to contribute to science [75], reinforce the commitment of the participant to accurately maneuver the instrumented boat and collect useful environmental data. We would expect that these motivational drivers could be also useful in enhancing patients’ adherence to rigid exercise regimens, which are often involved in rehabilitation treatments and perceived as tedious [83, 84].

Our approach builds on the clinical advancements enabled by RMT in evaluating motion performance [66] toward an affordable, versatile, and portable technology for rehabilitation. While RMT often entails the use of complex and expensive devices that can only be accessed in specialized facilities, the proposed framework requires minimal clinical and technical infrastructure, which should facilitate rapid and simple implementations in diverse environments, including the patients’ home [85]. In such home settings, the patients could drive the miniature instrumented boat from their personal computers and contribute to citizen science remotely. Differently from the testing scenario presented in this study, a real rehabilitation session could be programmed by asking participants to drive the sensor-equipped boat through several target locations in the canal, and requiring a time duration comparable with traditional rehabilitation sessions. Such a design will afford the delivery of the requisite quantity (duration and frequency) and quality (task-specificity) of treatment that is needed for physical rehabilitation [86, 87]. The scalability of the tested platform, enabled by the use of motion capture rather than a mechatronic device, might allow to rapidly re-map the gesture under recommendation of specialized clinicians, and include subjects in need of various typologies of physical exercise or rehabilitation.

Our approach might particularly benefit patients with a motor impairment of appropriated severity, who can exhibit sufficient abilities to attempt the citizen science task. In such case, future works should involve rehabilitation outpatients after stroke and use the results of this study with healthy subjects as a baseline. Patients with disabilities too severe would likely require more traditional treatments or treatments with robotic devices to provide haptic assistance [88]. In particular, we envisage including patients, based on clinical evaluation of their impairment by medical specialists. The clinician’s case-by-case opinion, grounded both on the clinical status and risk evaluation, will be the final driver for inclusion. However, scores obtained via clinical motor impairment scales widely adopted in stroke evaluation, such as the Fugl-Mayer score [89], are likely to offer a first metric for the inclusion of patients. Specifically, adult patients, with a Fugl-Mayer score <50/66, sufficient cognitive skills, and able to sit and stand independently are expected to benefit from the platform proposed here.

Due to the high scalability of our natural user interface, this solution may be adapted to a different rehabilitation approach for post-stroke hemiparesis, based on the bilateral manipulation of objects [90]. Modification of the implemented gestures to bilateral, under clinicians’ supervision, would adapt the tested solution to patients with a moderate level of impairment severity, who can leverage the strength of the unaffected side to help the affected one, activating motor synergies between limbs [91].

Similar to systems used in RMT, the Kinect platform allows for evaluating an array of motion performance measures which could aid in assessing the patient recovery, without the continuous presence and direct supervision by a physical therapist, who could simultaneously supervise several patients at the same time. The simplicity of the setup tested in this work, only based on a low-cost markerless motion tracker, allows for its integration with a set of other sensors typically adopted in the evaluation of motor impairment.

In an extended version of the proposed paradigm, the inclusion of recently developed wearable force or torque sensors [92, 93] may provide a more comprehensive and valuable assessment of motor impairment, not only based on kinematics. By further integrating other devices, future incarnations of the proposed platform could extend exercise and evaluation indices to the lower limb as well. The inclusion of recent wearable solutions that have been introduced and validated for the lower limb [94–97], may extend the evaluation of kinematics and dynamics to the lower extremity. This is a crucial body region for a recovering patient’s autonomy, and the possibility of including inertial and force sensors may considerably empower the effectiveness of the Kinect platform.

## Supporting information

S1 Table Processed dataset. The table contains classification data of all anonymous participants (gender, used device, water quality data collection), along with time of completion of the task, kinematic indices calculated from raw data, and survey answers (Q4 to Q11).
(XLSX)

S1 Folder Kinect raw data. Kinect raw data of each participant in the two kinect groups (KC and KNC). Data of each participant are saved in a binary file. Trajectories of points have been extracted form the binary file and saved in a text file.
(ZIP)
